# Identifying the Gene Regulatory Network of the Starvation-Induced Transcriptional Activator Nla28

**DOI:** 10.1128/jb.00265-22

**Published:** 2022-11-30

**Authors:** Muqing Ma, Anthony G. Garza, David J. Lemon, Eduardo A. Caro, Linnea Ritchie, Charles Ryan, Victoria M. Spearing, Kimberly A. Murphy, Roy D. Welch

**Affiliations:** a Department of Biology, Syracuse University, Syracuse, New York, USA; Geisel School of Medicine at Dartmouth

**Keywords:** enhancer-binding proteins, σ^54^ promoters, biofilms, transcriptional activators, fruiting body development

## Abstract

Myxococcus xanthus copes with starvation by producing fruiting bodies filled with dormant and stress-resistant spores. Here, we aimed to better define the gene regulatory network associated with Nla28, a transcriptional activator/enhancer binding protein (EBP) and a key regulator of the early starvation response. Previous work showed that Nla28 directly regulates EBP genes that are important for fruiting body development. However, the Nla28 regulatory network is likely to be much larger because hundreds of starvation-induced genes are downregulated in a *nla28* mutant strain. To identify candidates for direct Nla28-mediated transcription, we analyzed the downregulated genes using a bioinformatics approach. Nine potential Nla28 target promoters (29 genes) were discovered. The results of *in vitro* promoter binding assays, coupled with *in vitro* and *in vivo* mutational analyses, suggested that the nine promoters along with three previously identified EBP gene promoters were indeed *in vivo* targets of Nla28. These results also suggested that Nla28 used tandem, imperfect repeats of an 8-bp sequence for promoter binding. Interestingly, eight of the new Nla28 target promoters were predicted to be intragenic. Based on mutational analyses, the newly identified Nla28 target loci contained at least one gene that was important for starvation-induced development. Most of these loci contained genes predicted to be involved in metabolic or defense-related functions. Using the consensus Nla28 binding sequence, bioinformatics, and expression profiling, 58 additional promoters and 102 genes were tagged as potential Nla28 targets. Among these putative Nla28 targets, functions, such as regulatory, metabolic, and cell envelope biogenesis, were assigned to many genes.

**IMPORTANCE** In bacteria, starvation leads to profound changes in behavior and physiology. Some of these changes have economic and health implications because the starvation response has been linked to the formation of biofilms, virulence, and antibiotic resistance. To better understand how starvation contributes to changes in bacterial physiology and resistance, we identified the putative starvation-induced gene regulatory network associated with Nla28, a transcriptional activator from the bacterium Myxoccocus xanthus. We determined the mechanism by which starvation-responsive genes were activated by Nla28 and showed that several of the genes were important for the formation of a highly resistant cell type.

## INTRODUCTION

Bacteria typically have numerous transcription factors that activate and/or repress the transcription of genes important for development. In the soil bacterium Myxococcus xanthus, the early developmental pathway relies heavily on enhancer-binding proteins or EBPs (1, 2), which are transcriptional activators that help σ^54^-RNA polymerase initiate transcription at σ^54^-type promoters (3–5). A cascade of EBPs modulates changes in developmental gene transcription during sequential stages of early development (6). This study aimed to identify and characterize the developmental gene targets of an early-functioning EBP called Nla28.

During development, M. xanthus forms a biofilm that contains a mat of rod-shaped cells known as peripheral rods (7, 8), and multicellular fruiting bodies that house about 100,000 dormant and stress-resistant spores (9). Starvation triggers the developmental process, and much is known about the subsequent morphological changes that yield spore-filled fruiting bodies. Cells migrate into aggregation centers, the aggregates become larger as a result, and they eventually develop the dome-shaped appearance of a fruiting body with rod-shaped cells in the newly formed fruiting bodies differentiated into spherical cells. The spherical cells mature into stress-resistant spores.

In standard developmental assays, at least two starvation-induced signaling events must occur before cells begin building fruiting bodies. The first event is the RelA-mediated accumulation of the intracellular starvation signal (p)ppGpp (10, 11). The second event, which depends on relatively high levels of (p)ppGpp (11), is the accumulation of an extracellular signal called A-signal. A-signal is a cell-density signal composed of amino acids and perhaps peptides (12–14). Functionally, it is hypothesized that (p)ppGpp accumulation indicates cells are starving, and the subsequent accumulation of A-signal indicates that enough starving cells are present to build a fruiting body.

Our previous work linked four of the 53 M. xanthus EBPs to the accumulation of early developmental signals (15–19). The EBP Nla28, which is the focus of this study, was linked to A-signal production via extracellular complementation assays (15). Nla28 is a response regulator-type EBP that partners with the membrane-bound histidine protein kinase Nla28S to form a two-component signal transduction system (20, 21). Two pieces of data led to the suggestion that the Nla28S/Nla28 signal transduction system might respond to A-signal in addition to being important for A-signal production (21). First, *nla28S* and *nla28* form a two-gene operon and Nla28 is involved in autoregulation (6). Second, A-signal is important for full developmental expression of the *nla28S* gene and, presumably, the *nla28S*-*nla28* operon (20).

In addition to being connected to A-signal production, *nla28* is known to be expressed early in development and is important for the expression of early developmental genes. DNA microarray data showed that inactivation of *nla28* impairs or abolishes the expression of many genes that are induced 1 to 2 h poststarvation (6). This finding led to the suggestion that the Nla28S/Nla28 signal transduction system targets some of the starvation-associated or stress-responsive genes (19, 21). Indeed, the results of a recent study suggest that Nla28 directly modulates the activities of three natural product promoters during the transition into the stationary-phase and during development. Both events are associated with nutrient depletion (22).

Although Nla28 is important for the expression of hundreds of M. xanthus developmental genes and the developmental process (6, 15), only two classes of developmental genes have been linked to direct Nla28-mediated regulation. These are the regulatory genes and natural product-associated genes (6, 22). The first goal of this study was to determine how Nla28 identified its target promoters. The second goal was to use this information to identify the larger network of Nla28-targeted promoters, with an emphasis on promoters that showed substantial developmental regulation. The third and final goal was to analyze genes under direct Nla28 control to better understand the function of Nla28.

Because EBPs modulate transcription at σ^54^ promoters (23, 24), our search for direct Nla28 targets focused on known or putative σ^54^ promoters. Namely, we searched for known or putative σ^54^ promoters upstream of genes that showed substantial developmental regulation and at least 2-fold downregulation in a *nla28* mutant. Further analyses suggested that the candidate σ^54^ promoters and genes were indeed *in vivo* targets of Nla28 and that Nla28 used tandem 8-bp sequences for promoter binding. Our results also indicated that one or more genes in each Nla28 target loci were important to produce stress-resistant spores during starvation-induced development. Additional candidates for direct Nla28 regulation were identified using the consensus Nla28 binding sequence. The Nla28 regulon was likely to be well over 100 genes. Known and predicted functions of Nla28 target genes are discussed in the context of M. xanthus development.

## RESULTS

### A set of potential developmental promoter targets directly regulated by Nla28 were identified.

In our initial studies, we aimed to identify and characterize a collection of developmentally regulated promoters that were likely to be under direct transcriptional control of Nla28 *in vivo*. Our search for direct targets of Nla28 started with a set of M. xanthus genes that showed substantial changes in expression during development. Between any two time points in the developmental time course, expression of the genes increased by at least 2-fold (our previously published DNA microarray data on Gene Expression Omnibus, accession number GSE13523) (6). We then searched for genes that showed at least a 2-fold decrease in expression when comparing any developmental time point in the *nla28* mutant and wild-type cells (6). Fifty-one genes with substantial developmental regulation met this criterion and, hence, were classified as Nla28-dependent. Notably, the genes in the *actB*, *nla6*, and *nla28* operons were not included in this analysis because previous work showed that certain regions of the *act* and *nla* promoters were bound by the Nla28 DNA binding domain (Nla28-DBD). These promoters were already known to be good candidates for direct *in vivo* regulation by Nla28 (6). These promoters were, however, included in subsequent studies to identify the Nla28 promoter binding sites.

**FIG 1:**
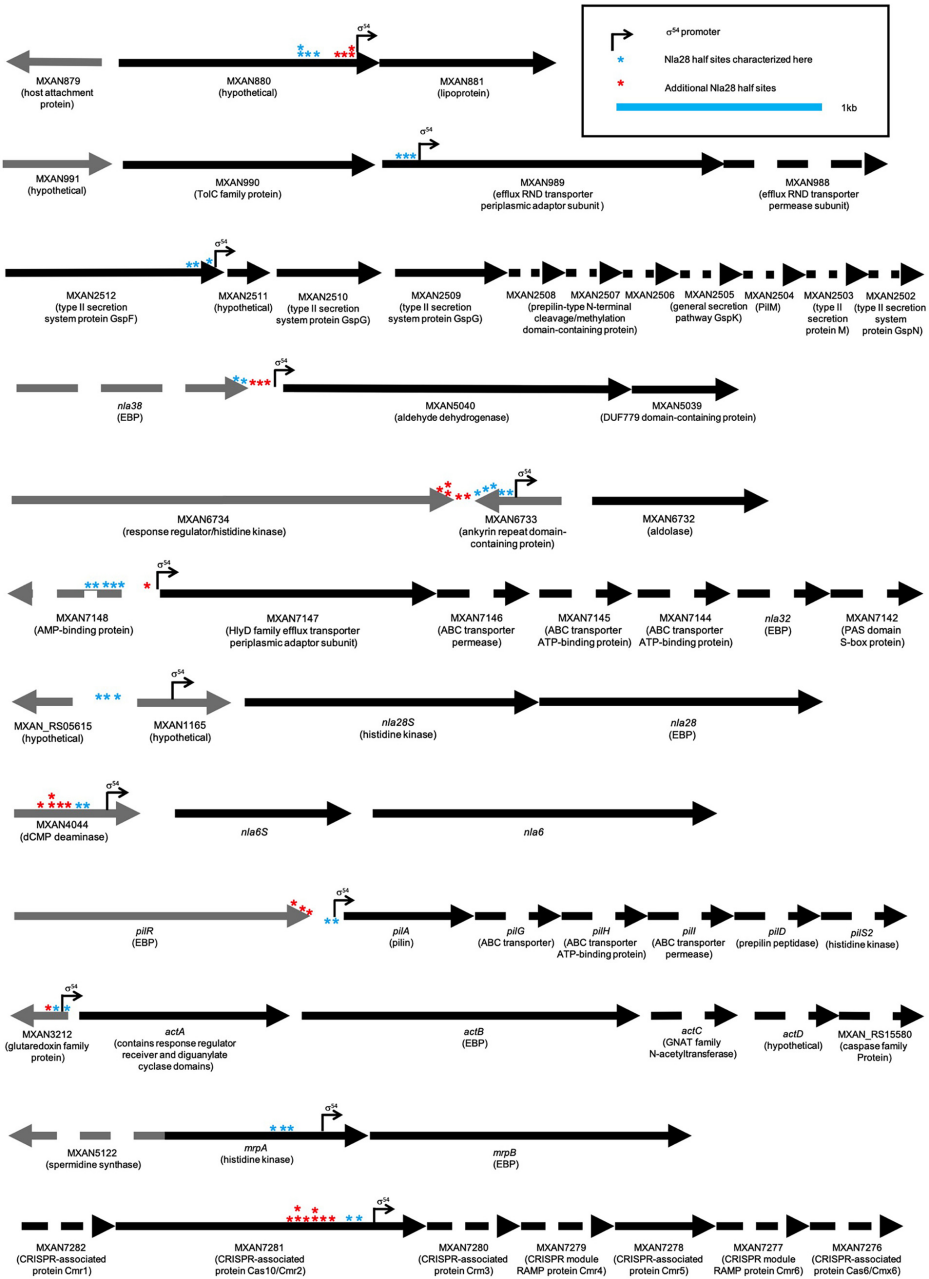
Locations of characterized developmental promoter targets of Nla28. Genes are represented by black (Nla28 targets) or gray (other loci) arrows oriented in the direction of transcription. Dashed arrows represent relatively large genes that are not drawn to scale. Bent arrows represent known or putative σ^54^ promoters. Blue asterisks denote Nla28 half-binding sites analyzed here. Red asterisks denote additional putative Nla28 half-binding sites identified in the promoter regions.

The DNA region upstream of the 51 Nla28-dependent genes was scanned for putative σ^54^ promoters using the algorithm developed for PromScan, a bioinformatics tool that identifies potential σ^54^-RNA polymerase binding sites using conserved nucleotides in the −12-bp and −24-bp regions of σ^54^ promoters (25). Because of the distant location (in the primary DNA structure) of the σ^54^ promoter elements of the *nla6* and *nla28* operons, we searched for putative σ^54^ promoters in the 500-bp region upstream of each Nla28-dependent developmental gene. Intergenic and intragenic regions were evaluated because previous mutational analyses placed the putative σ^54^-RNA polymerase binding sites in the *actB*, *nla6*, and *nla28* promoters in intragenic regions (Fig. 1). Notably, previous PromScan trial runs with known σ^54^ promoters and non-σ^54^ promoters yielded a false-positive rate of about 4% and a false-negative rate of about 19% (6). Therefore, we anticipated similar false assignments in our search.

Of the 51 Nla28-dependent genes analyzed as described above, only nine had a putative σ^54^-RNA polymerase binding site in the 500-bp region. These nine promoters, which included the previously characterized promoter in the *pilA* locus (26) and the promoters of the *actB*, *nla6*, and *nla28* operons, were all considered candidates for direct *in vivo* regulation by Nla28 (Table 1).

**TABLE 1:** Characterized developmental promoter targets of Nla28

Locus	Genes	Notable functions or putative functions of the protein product(s)	Activation time of development^*a*^	Putative σ^54^-RNA polymerase binding site (−12-bp and −24-bp promoter regions)^*b*^	Putative Nla28 half-binding sites^*d*^
*actB*	4	ActB EBP and ActA signal transduction protein	4 hours	**TGGCAC**A-N4-**TTGCT**^*c*^	**C**G**CCGC**G**G**-N54-**CTGC**C**CAG**
*nla6*	2	Nla6S histidine kinase and Nla6 EBP	1 hour	**TGG**TG**CG**-N4-G**TG**T**T**^*c*^	**CTGCG**TG**G**-N9-AC**GCGGAG**
*nla28*	2	Nla28S histidine kinase and Nal28 EBP	1 hour	**TGG**AG**CG**-N4-C**TGCT**^*c*^	G**TG**G**GGAG**-N3-**CTCCGCAG**-N25-**CTCC**C**CAG**
*mrpB*	1	MrpB EBP	1 hour	**TGGC**C**C**A-N4-C**TGCT**	**C**G**G**T**GCAG**-N47-**CTCC**T**C**G**G**-N9-**C**G**G**T**GCAG**
*pilA*	6	Type IV pili biogenesis	-	**TGGCA**T**G**-N4-G**TGCT**	G**TGCGCA**C-N10-A**TGCG**T**AG**
MXAN881	1	Putative lipoprotein	1 hour	**TGGC**GT**G**-N4-G**TGCT**	G**TCCGG**C**G**^*e*^-N10- **CTCC**C**GA**T-N37-**CTC**A**G**A**AG** or **C**G**CCGGA**T^*e*^-N4-**CTCC**C**GA**T-N37-**CTC**A**G**A**AG**
MXAN989	2	Copper/silver cation efflux proteins (CusA and CusB)	1 hour	CC**GCACG**-N4-**TTGCT**	**C**A**GCGG**C**G**-N6-**C**G**CCGC**T**G**-N10-**C**C**C**T**GGAG**
MXAN2511	10	Type II protein secretion systems	1 hour	G**GGC**G**CG**-N4-**TTGCA**	**CTCCGG**CC-N14-**CTC**AC**CAG**-N67-**CTCC**T**CA**T
MXAN5040	2	Aldehyde dehydrogenase	-	**TGGCACG**-N4-C**TGCT**	TG**GCGCAG**-N12-**CTG**G**GCA**T
MXAN6732	1	Class II aldolase	12 hours	C**GGCA**T**G**-N4-**TTGC**G	**CTC**A**GC**C**G**-N36-G**TCCGC**C**G**-N17-T**TGCGCAG**-N19-G**TC**A**GCAG** or **CTCCGC**CA-N28-G**TCCGC**C**G**-N17-T**TGCGCAG**-N19-G**TC**A**GCAG**
MXAN7147	2	RND family efflux transporter and ABC-type efflux transport protein	1 hour	**TGGCACG**-N4-**T**C**GCT**	**CTCC**C**C**CG-N8-**C**C**GCGC**T**G**-N41-G**TCCG**T**AG**-N21-G**TC**A**GCAG**-N6-**CTC**G**GG**C**G**
MXAN7280	5	Putative CRISPR-associated proteins	1hour	**TGGCAC**C-N4-**T**C**GC**G	**CTG**T**GGA**C-N52-**CTG**TC**GAG**

a The indicated times are based on the qPCR data presented here or on previously published microarray data (6). The dashes are indicating that the activation time of corresponding genes pilA and MXAN5040 during development were not confirmed in this study or on previously published microarray data.

b The bold and underlined nucleotides match the nucleotides in the consensus σ^54^-RNA polymerase binding sequence, which is TGGCACG-4N-TTGC(T/A). N4 indicates any four nucleotides.

c The −12 and −24 regions of the putative σ^54^ promoter were characterized via mutational analysis.

d The bold and underlined nucleotides match the nucleotides in the Nla28 consensus half-binding site (CT[C/G]CG[C/G]AG). N indicates the number of nucleotides in the spacer region between half-sites.

e Overlapping sites found in the same promoter region.

**FIG 2:**
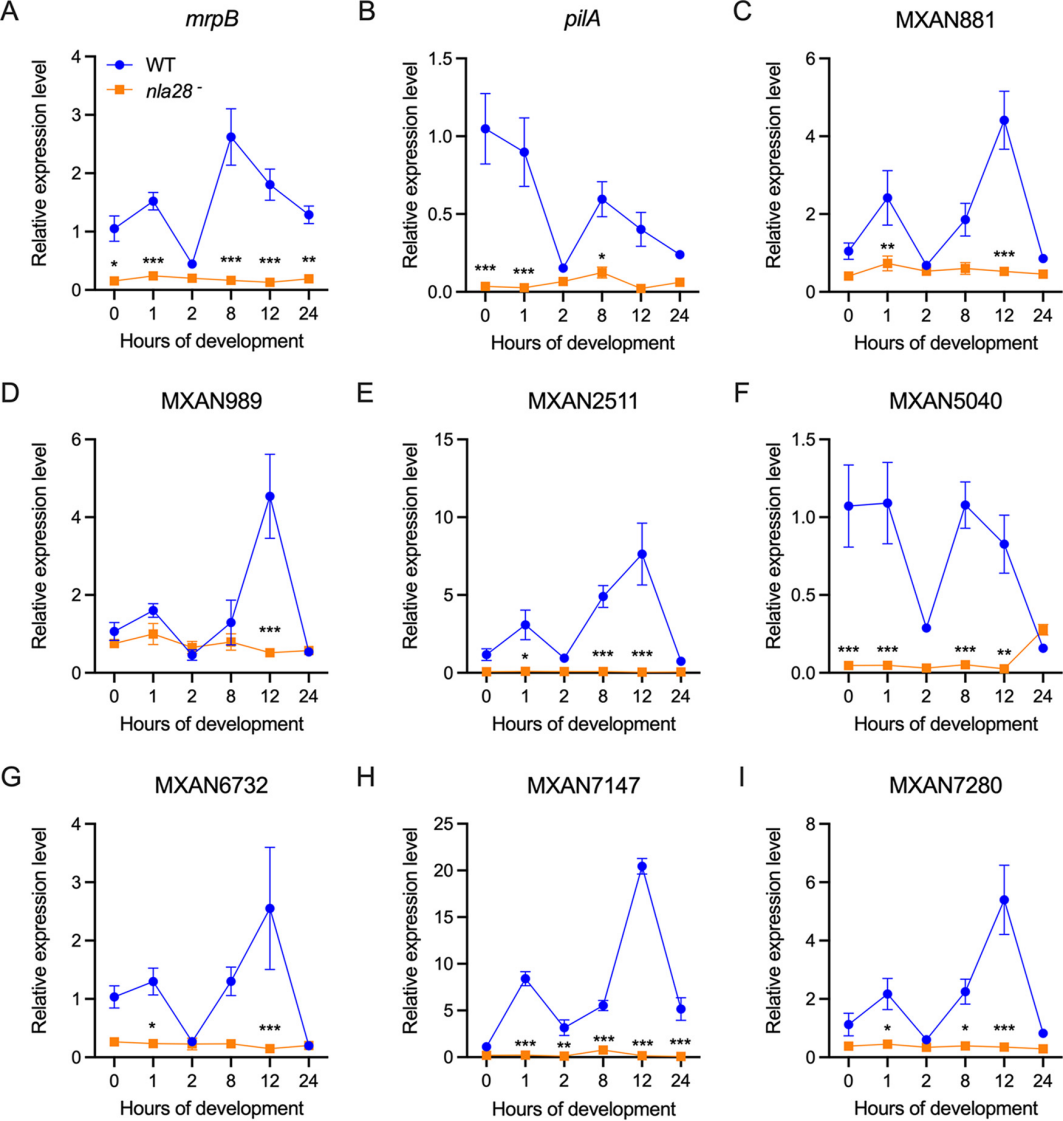
Quantitative real-time PCR measurement of the developmental expression levels and patterns of Nla28 target genes in wild-type and *nla28* mutant strains. The developmental mRNA levels of nine Nla28 target genes in wild-type (WT) and *nla28* mutant (*nla28*−) strains were determined using qPCR. Wild-type and *nla28* mutant cells were harvested at 0, 1, 2, 8, 12, and 24 h of development for RNA isolation and qPCR analysis. N = 3 technical replicates of pooled RNA samples at each time point. Error bars are standard deviations of the means. The data were analyzed using two-way analysis of variance (ANOVA) and Tukey’s multiple comparisons *post hoc* tests; ***, *P* < 0.001; **, *P* < 0.01; *, *P* < 0.05 for mRNA levels in *nla28* mutant versus wild-type cells.

To confirm the previously published DNA microarray data, which indicated that the nine newly identified promoters were Nla28 dependent *in vivo*, we used quantitative PCR (qPCR). We examined whether the developmental expression of one gene downstream of each promoter was downregulated in a *nla28* mutant relative to the wild-type strain. We compared the mRNA levels of the genes in the *nla28* mutant and wild-type cells at various developmental time points. The developmental expression patterns of the genes in the wild-type and *nla28* mutant cells are shown in Fig. 2, and a summary of how the *nla28* mutation impacted their developmental expression is summarized in Table S3 in Supplemental File 1.

Seven of the mRNAs that we examined showed significant increases during the development of wild-type cells. Peak developmental expression levels ranged from about 2.5- to 20.5-fold higher than the baseline vegetative (0 h) levels (Fig. 2A, C, E, G, and I). In contrast, the levels of *pilA* mRNA did not rise above the vegetative baseline during the wild-type developmental time course (Fig. 2B). However, our qPCR analysis lacked the developmental time point (6 h) at which *pilA* mRNA levels peaked in the microarray studies (6). Perhaps this resulted in the qPCR’s failure to detect a burst of *pilA* mRNA in developing cells. The qPCR analysis of wild-type cells also failed to detect an increase in MXAN5040 mRNA above the vegetative baseline (Fig. 2F), a finding that agreed with the results of the microarray analysis (6). This led us to examine the MXAN5040 mRNA expression pattern more closely, which revealed why MXAN5040 was classified as a locus that showed a substantial increase in expression during development. A locus was defined as such when it showed a 2-fold expression increase between any two time points in the developmental time course. MXAN5040 mRNA levels increased more than 2-fold between 2 and 8 h of development but never rose above vegetative levels due to the sharp decrease in expression between 1 and 2 h (Fig. 2F).

In the *nla28* mutant, the vegetive baseline levels of mRNAs for three genes, *mrpB*, *pilA*, and MXAN5040, were reduced (Fig. 2A to I), suggesting that Nla28 was important for the expression of these mRNAs in vegetative cultures and supporting the previous assertion that Nla28 was not a fruiting body-specific regulator (22). As for the mRNA levels in developing *nla28* mutant cells, expression was substantially lower at the wild-type peak and at least one time point but typically at multiple time points in development (Table S3 in Supplemental File 1 and Fig. 2A to I). These findings indicated that Nla28 was important for the normal expression of the mRNAs during development.

### Analyzing the chromosomal regions containing putative Nla28 target promoters.

The locations and sequences of the putative σ^54^-RNA polymerase binding sites in the −12-bp and −24-bp regions of the 9 newly identified promoters are shown in Fig. 1 and Table 1, respectively. The locations of the putative −12-bp and −24-bp regions of the *actB*, *nla6*, and *nla28* promoters were also shown because previous data indicated that Nla28 was bound to these promoters and that they were likely to be σ^54^-type promoters (6, 19). It is noteworthy that 9/12 of the putative σ^54^-RNA polymerase binding sites or core σ^54^ promoter regions were located within genes and not in intergenic sequences. This finding is consistent with previous studies that placed many core σ^54^ promoter regions in the coding sequences of M. xanthus genes (6, 16, 19, 22) and raised the possibility that intragenic σ^54^ promoters might be common in M. xanthus and bacteria in general. As in previous analyses of M. xanthus σ^54^ promoters, some of the intragenic −12-bp and −24-bp regions were in the protein-coding sequence of an upstream gene (upstream promoters) and some were in the protein-coding sequence of one gene in an operon (internal promoters) (22). The implications of these findings are addressed in the Discussion.

### Promoter fragments positive for *in vitro* Nla28-DBD binding had similar 8-bp repeats.

**FIG 3:**
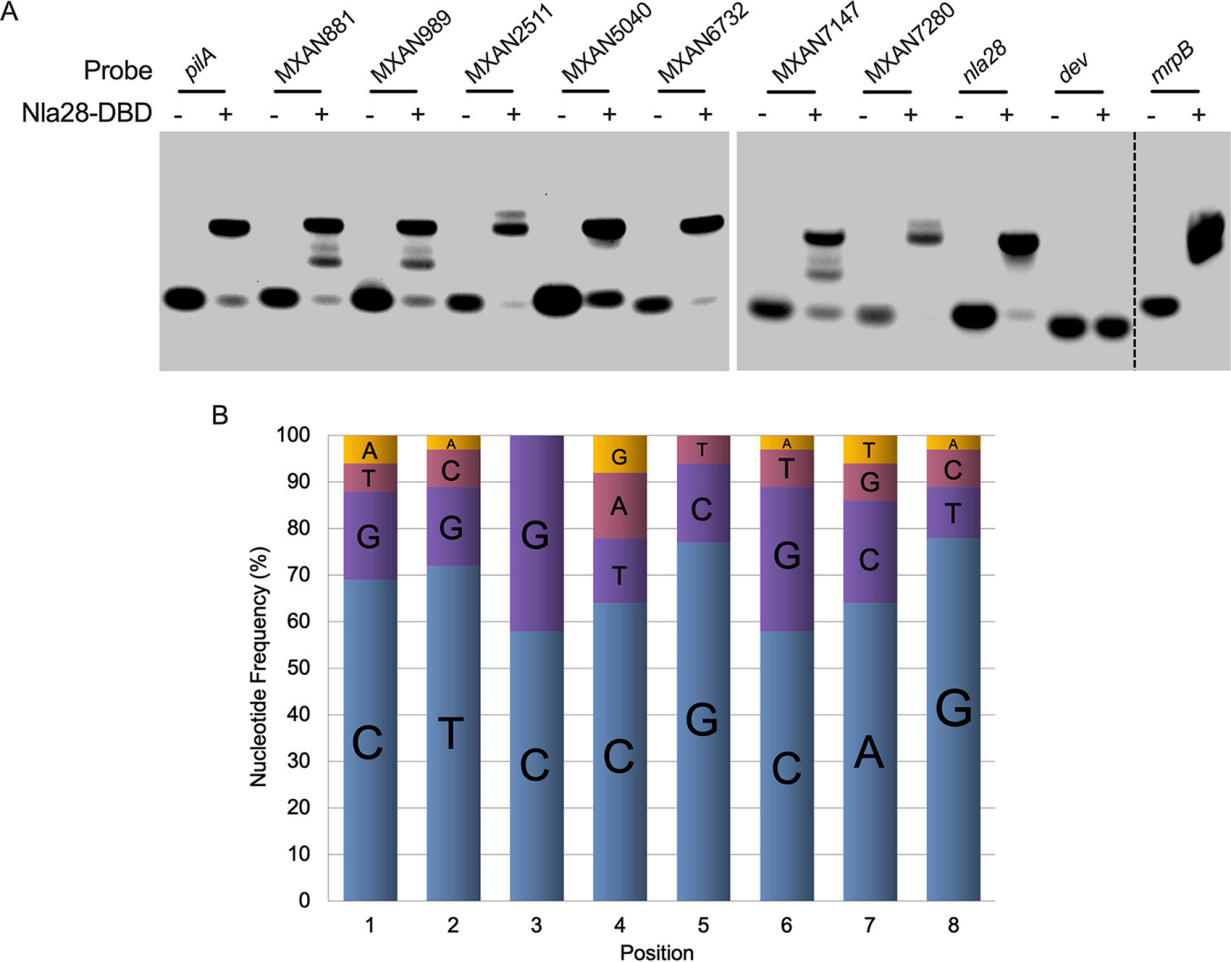
Electrophoretic mobility shift assays with Nla28-DBD and fragments of putative target promoters. (A) Purified Nla28-DBD binds to fragments of the 9 newly identified target promoters. EMSAs performed with purified Nla28-DBD and a *pilA*, MXAN881, MXAN989, MXAN2511, MXAN5040, MXAN6732, MXAN7147, MXAN7280, *nla28* (positive control), *dev* (negative control) or *mrpB* promoter fragment containing putative Nla28 binding sites. Binding reactions were performed with (+) or without (−) 2 μM purified Nla28-DBD and 5 ng of Cy5 5′ end-labeled promoter fragments in a total volume of 10 μL. Similar 8-bp sequences (putative Nla28 half-binding sites) were found in all promoter fragments that were positive for Nla28 binding. (B) Nucleotide frequency at each position of the 8-bp sequence. The consensus 8-bp sequence or consensus Nla28 half-binding site derived from the frequency data was CT(C/G)CG(C/G)AG.

Electrophoretic mobility shift assays (EMSAs) were used to examine whether Nla28-DBD was capable of binding to at least one DNA fragment in the nine newly identified promoter regions, where 180-bp to 220-bp DNA fragments flanking the putative σ^54^-RNA polymerase binding sites in the −12-bp and −24-bp regions upstream of Nla28 target genes were used. Nla28-DBD was consistently positive for binding to at least one fragment of each promoter region. All the promoter fragments except the negative control *dev* promoter fragment, which is a fragment of a non-σ^54^ promoter, produced at least one shifted complex (Fig. 3A). The positive control, which is a fragment of the *nla28* promoter, also produced a shifted complex as predicted (Fig. 3A).

When we searched the promoter fragments that were positive for Nla28-DBD binding, we identified similar 8-bp sequences (Table 1). Each promoter fragment tested here contained at least two but often 3 to 5 of these 8-bp sequences (Table 1), as did the *actB*, *nla6*, and *nla28* promoter fragments, which were previously shown to be positive for Nla28-DBD binding (6, 19). Because EBP dimers typically bind to tandem repeat sequences or tandem half-binding sites (23, 24), we speculated that at least two of the 8-bp sequences in each promoter fragment served as Nla28-DBD half-binding sites. We also speculated that Nla28-DBD might be able to form different binding complexes when the promoter fragments have more than one half-site pair. This might explain why promoter fragments, such as MXAN881, MXAN989, and MXAN7147, yielded more than one shifted complex in the EMSAs (Fig. 3A). We should note that many of the promoter regions contained a second putative cluster of Nla28 half-sites (Fig. 1). We identified these sites using the consensus Nla28 half-binding site 5′-CT(C/G)CG(C/G)AG-3′ (Fig. 3B).

In a subsequent search for additional Nla28 targets, the M. xanthus genome sequence (16) was scanned for close matches to the consensus Nla28 half-binding site (Fig. 3B). We looked for 8-bp sequences with no more than two mismatches relative to the consensus Nla28 half-binding site. Because EBP dimers bind to DNA, we also looked for potential Nla28 half-sites arranged in tandem. Tandem sites were defined as putative Nla28 half-sites separated by no more than 25 bp, which was the mean number of base pairs between putative Nla28 half-sites in the 12 promoter regions that we characterized. We decided to use this relatively conservative upper limit on the putative spacer region between repeats/putative half-binding sites because all the Nla28 binding sites characterized by mutational analysis, except for the putative binding site in the *actB* promoter, had shorter spacer regions (6, 22). Therefore, we felt more confident that the tandem repeats that were selected might be Nla28 binding sites. Additionally, we also searched for putative σ^54^-RNA polymerase binding sites located within 500-bp downstream of the tandem sequences. The locations and sequences of putative Nla28 half-binding sites and σ^54^-RNA polymerase binding sites are shown in Table S4 in Supplemental File 2.

### Two 8-bp sequences in the *actB* promoter and the *nla28* promoter were crucial for *in vitro* Nla28-DBD binding.

**FIG 4:**
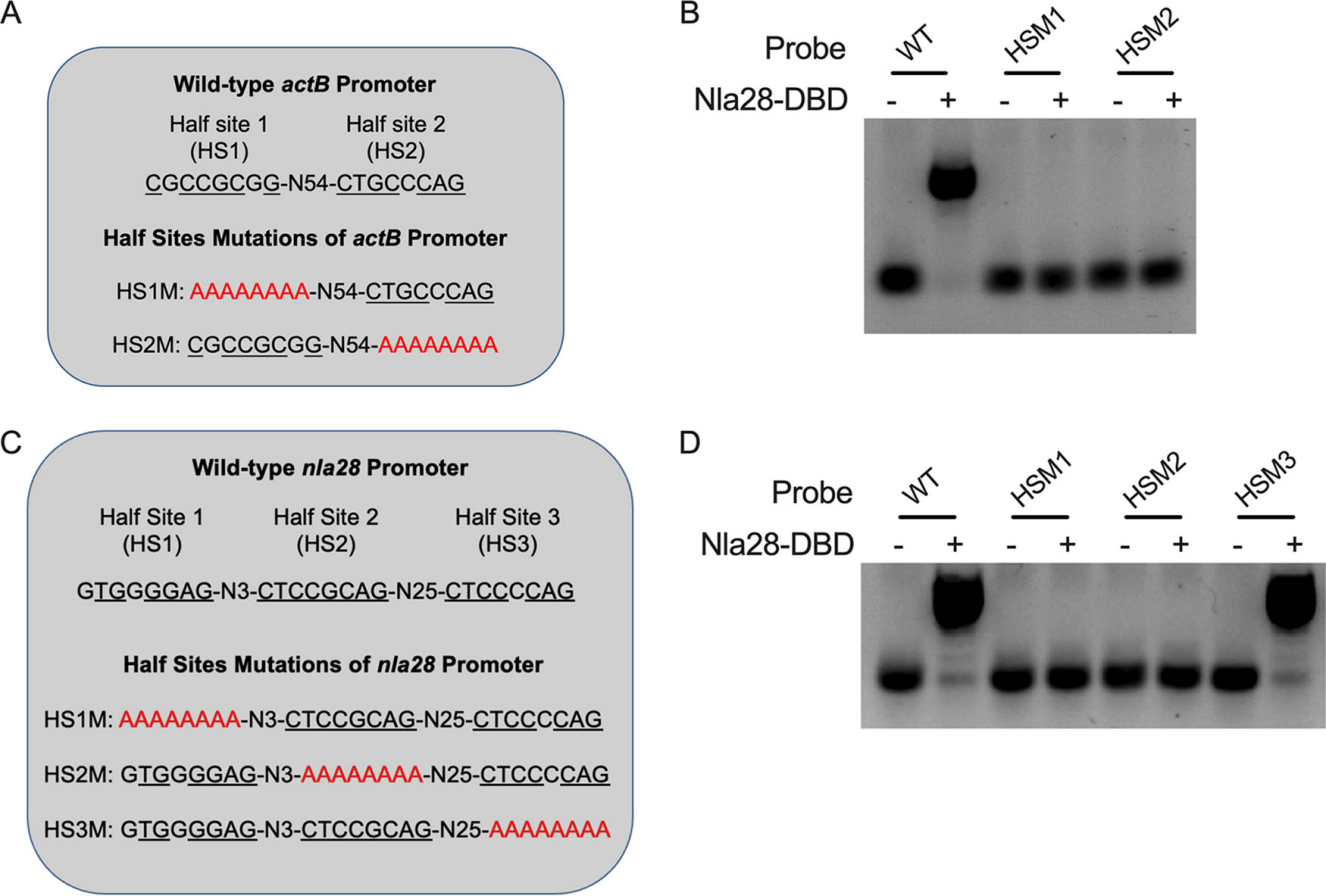
Electrophoretic mobility shift assays with Nla28-DBD and wild-type or mutant fragments of the *actB* and *nla28* promoters. (A) Two 8-bp sequences, half-site 1 (HS1) and half-site 2 (HS2), that closely matched the consensus Nla28 halfsite-binding were identified in the wild-type (WT) *actB* promoter fragment. Underlined sequences represent nucleotides that matched the consensus Nla28 half-binding site. *actB* promoter fragments (*actB* HS1M and HS2M) carrying mutations in these putative Nla28 half-binding sites were generated for *in vitro* Nla28-DBD binding analysis. (B) EMSAs were performed with (+) or without (−) 2 μM purified Nla28-DBD and 5 ng of a Cy5 5′ end-labeled *actB* promoter fragment containing two WT Nla28 half-binding sites, *actB* HS1M or *actB* HS2M, in a total volume of 10 μL. (C) Three 8-bp sequences (HS1, HS2, and HS3) that closely matched the consensus Nla28 half-binding site were identified in the wild-type *nla28* promoter fragment. Underlined sequences represent nucleotides that matched the consensus Nla28 half-binding site. *nla28* promoter fragments (*nla28* HS1M, HS2M, and HS3M) carrying mutations in these putative Nla28 half-binding sites were generated for *in vitro* Nla28-DBD binding analysis. (D) EMSAs were performed with (+) or without (−) 2 μM purified Nla28-DBD and 5 ng of Cy5 5′ end-labeled *nla2B* promoter fragment containing three WT Nla28 half-binding sites, *nla28* HS1M, *nla28* HS2M, or *nla28* HS3M, in a total volume of 10 μL.

Because all the promoter fragments that were positive for Nla28-DBD binding in EMSAs contained similar 8-bp sequences, we investigated if these sequences are important for Nla28-DBD binding. We initially focused on the *actB* promoter fragment, which has only two of the 8-bp sequences (i.e., two putative half-sites) for the presumed binding of a Nla28-DBD dimer. Three *actB* promoter fragments were generated for the *in vitro* Nla28-DBD binding analysis. One of the *actB* promoter fragments had wild-type half-sites 1 and 2. One fragment had a wild-type half-site 1 and a half-site 2 that was converted to all adenine (A) nucleotides, and one fragment had wild-type half-site 2 and a half-site 1 that was converted to all A nucleotides (Fig. 4A). EMSAs were performed with 2 μM Nla28-DBD and 5′ Cy5-labeled wild-type or mutant *actB* promoter fragments. Nla28-DBD was capable of binding to the *actB* promoter fragment carrying two wild-type half-sites as in previous EMSAs. However, no binding was detected when either half-site 1 or half-site 2 was converted to all A nucleotides (Fig. 4B). This finding is consistent with our proposal that the two 8-bp sequences served as half-binding sites for Nla28-DBD dimers and that both sites are important for Nla28-DBD binding.

In the next experiment, we examined Nla28-DBD binding to the *nla28* promoter fragment, which contained three 8-bp sequences or potential Nla28-DBD half-binding sites. Four *nla28* promoter fragments were generated for the *in vitro* binding assays. One of the *nla28* promoter fragments contained wild-type half-sites 1 to 3, and each of the remaining *nla28* promoter fragments contained two wild-type half-sites and one-half-site converted to all A nucleotides (Fig. 4C). EMSAs were performed with 2 μM Nla28-DBD and 5′ Cy5-labeled wild-type or mutant *nla28* promoter fragments. Nla28-DBD binding was detected when the *nla28* promoter fragment contained all wild-type half-sites, as previously noted. Nla28-DBD binding was also detected when the *nla28* promoter fragment contained wild-type half-sites 1 and 2, and a half-site 3 that was converted to all A nucleotides. However, no Nla28-DBD binding was detected when half-site 1 or half-site 2 was converted to all A nucleotides, even though the remaining two half-sites were wild-type (Fig. 4D). Thus, it seemed that half-sites 1 and 2, but not half-site 3, in the *nla28* promoter were crucial for *in vitro* Nla28-DBD binding. This was not the predicted result based solely on a putative half-site similarity to the consensus because half-sites 1, 2, and 3 had 2, 0, and 1 mismatch(es), respectively, relative to the consensus Nla28 half-binding site (Fig. 4C and Table 1). Perhaps some nucleotide positions in a half-site were more important for Nla28-DBD binding than others and/or the spacing between one half-site, and its neighboring half-site was important for the efficient binding of Nla28-DBD *in vitro*. Of course, in an *in vivo* setting, it is likely that Nla28’s conformation and oligomerization states, which are presumably influenced by Nla28S-mediated phosphorylation (20), and perhaps the intrinsic curvature of the promoter region would also influence binding.

### Mutations in putative Nla28 binding sites substantially reduced promoter activity in developing cells.

**FIG 5:**
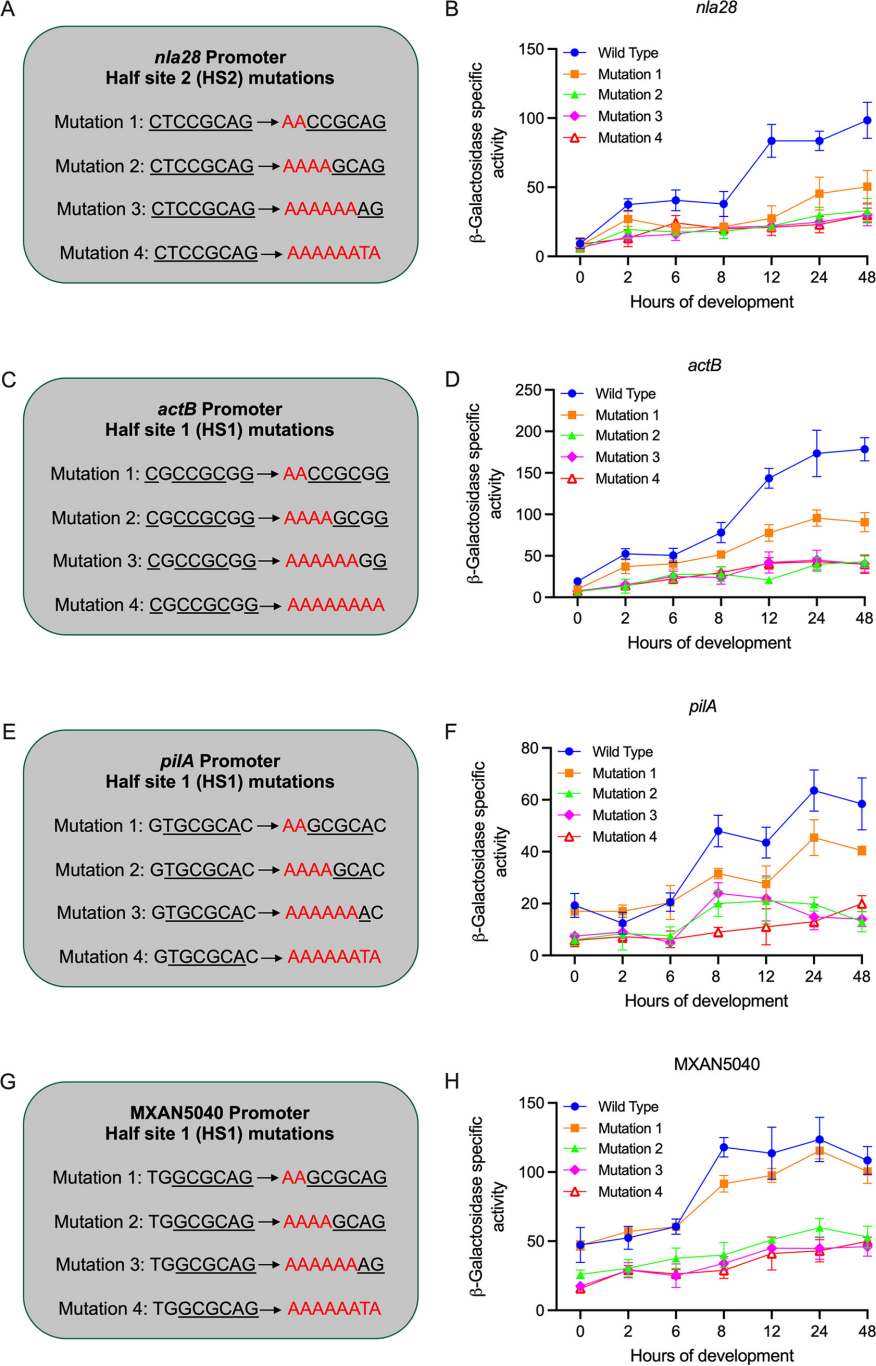
*In vivo* activities of wild-type and mutant promoter targets of Nla28. (A, C, E, and G) *nla28*, *actB*, *pilA*, and MXAN5040 promoter fragments with a 2-bp substitution (mutation 1), 4-bp substitution (mutation 2), 6-bp substitution (mutation 3), or 8-bp substitution (mutation 4) in Nla28 half-site 2 were generated by site-directed mutagenesis. Substituted nucleotides are shown in red. (B, D, F, and H) Wild-type and derivatives of the *nla28*, *actB*, *pilA*, and MXAN5040 promoters carrying mutation 1, mutation 2, mutation 3, or mutation 4 were cloned into a *lacZ* expression vector and transferred to the wild-type M. xanthus strain DK1622. *In vivo* activities of wild-type and mutant promoters were inferred from β-galactosidase-specific activities (defined as nanomoles of ONP produced per minute per milligram of protein) at various time points (0, 2, 6, 8, 12, 24, and 48 h) during development. N = 3 biological replicates at each of the indicated time points. Error bars are standard deviations of the means.

The *in vitro* binding and *in vivo* expression data presented here and in previous work (6) suggested that Nla28 directly regulated the nine newly identified promoters, as well as the *actB*, *nla6*, and *nla28* promoters. To further examine this idea, we selected four Nla28 target promoters for an *in vivo* mutational analysis. We started with fragments of the *nla28*, *actB*, *pilA*, and MXAN5040 promoter regions. Each fragment contained the putative σ^54^-RNA polymerase and Nla28 binding sites. These four promoters were selected because two of the promoters (*nla28* and *actB*) showed substantial developmental regulation (6), and two (*pilA* and MXAN5040) did not appear to be developmentally regulated based on qPCR (Fig. 2). Next, fragments with a 2-bp substitution, 4-bp substitution, 6-bp substitution, or 8-bp substitution in Nla28 half-sites 1 or 2 were generated (Fig. 5A to H). The native nucleotides were converted to A or thymine (T) nucleotides in all cases.

Wild-type and mutant *nla28* promoter fragments were cloned into pREG1727, a plasmid that creates transcriptional fusions between cloned promoters and the *lacZ* gene (27). The *lacZ* fusion plasmids were introduced into wild-type strain DK1622, and Kan^r^ isolates carrying a plasmid integrated at the Mx*8* phage attachment site in the M. xanthus chromosome were identified via PCR and analyzed via DNA sequencing. Strains carrying a wild-type or mutant promoter fragment were allowed to develop for various amounts of time and β-galactosidase assays were used to infer developmental promoter activities.

As shown in Fig. 5A and B, a 4-bp, 6-bp, or 8-bp substitution in putative Nla28 half-site 2 substantially reduced the peak *in vivo* activity of the *nla28* promoter relative to that of the wild-type. Similar results were observed with the *actB*, *pilA*, and MXAN5040 promoter fragments that contained a 4-bp, 6-bp, or 8-bp substitution in putative Nla28 half-site one, as shown in Fig. 5C to H. We also observed substantially reduced peak activities when the *nla28* and *actB* promoters contained a 2-bp substitution in putative Nla28 half-site 2 and 1, respectively (Fig. 5A to D). In contrast, a 2-bp substitution had only a slight impact on the peak activity of the *pilA* promoter (Fig. 5E and F) and no impact on the peak activity of the MXAN5040 promoter (Fig. 5G to H). In the case of the MXAN5040 promoter, it was not surprising that the two native nucleotides were not crucial for *in vivo* activity because they did not match the nucleotides in the consensus Nla28 half-binding site. The results of our *in vivo* and *in vitro* assays provided strong evidence that the 8-bp sequences that we identified were important for Nla28 binding and the *in vivo* activities of Nla28 target promoters.

### Nla28 target genes were important for development.

The results of our *in vitro* and *in vivo* expression studies indicated that the loci shown in Table 1 were likely to be *in vivo* targets of Nla28 during development. Four of the loci (*actB*, *nla6*, *nla28*, and *mrpB*) contained characterized EBP genes, and the corresponding EBPs were known to be key components in M. xanthus developmental regulatory pathways (15, 19, 28–30). Another locus that was characterized was *pilA*. This locus is important to produce type IV pili, social motility, and normal development (31–33).

To examine whether the remaining Nla28 targets listed in Table 1 were important for development, we generated an insertion in one gene in each locus. Specifically, we generated an insertion in the single gene or one gene in the operon located downstream of the putative core σ^54^ promoter element and then determined whether the insertions affected the formation of aggregates of cells and/or sporulation (Table 2). All of the insertions but MXAN6732 affected the timing of aggregate formation and some affected the final shape of aggregates, but none completely blocked the formation of aggregates. In addition, the insertions substantially reduced the number of sonication- and heat-resistant spores that were able to germinate into colonies, as indicated by the viable spore numbers. Interestingly, the insertions had relatively minor impacts on spore numbers. Spore number referred to the number of developing cells that were able to make the shape change (rod to spherical shape) associated with the early differentiation stage of sporulation (Table 2). Thus, it seemed that the insertions had a stronger impact on the maturation of spores into stress-resistant cells and/or on spore germination than on spore differentiation.

**TABLE 2:** Developmental phenotypes of wild-type and mutant cells

Strain^*a*^	Aggregation^*b*^	Normal shape^*c*^	Fruiting body sporulation^*d*^	
			Spore no. (%)	Viable spore no. (%)
DK1622	+	+	100 ± 12.6	100 ± 8.5
*nla28* ^*a*^	−	+	108.1 ± 7.6	2.8 ± 1.2
MXAN881^*a*^	−	+	72.2 ± 0.7	0.5 ± 0.5
MXAN989^*a*^	−	+	91.1 ± 5.0	<0.1
MXAN5040^*a*^	−	+	55.5 ± 7.3	1.2 ± 0.8
MXAN7147^*a*^	−	+	78.5 ± 8.1	7.9 ± 2.5
MXAN7279^*a*^	−	−	44.0 ± 7.8	5.4 ± 2.1
MXAN2510^*a*^	−	−	35.0 ± 8.6	<0.1
MXAN6732^*a*^	+	−	47.4 ± 3.7	6.3 ± 3.3

a The mutant strain of M. xanthus contained an insertion in the indicated gene.

b +, Formed aggregates as quickly as wild-type; −, aggregation was delayed.

c +, aggregates (after 5 days of development) had normal shapes; −, produced abnormally shaped aggregates.

d The mean values (±standard deviations) for the spore assays are shown as percentages of M. xanthus DK1622 (wild-type). Means (±standard deviations) derived from three independent experiments are shown.

### Insertions in some Nla28 target genes affected swarm expansion.

As noted above, one of the characterized targets of Nla28 was *pilA*, a gene that was important for type IV pili-based social motility, surface spreading, and normal development (31–33). Because many of the remaining Nla28 targets had yet to be tested for roles in M. xanthus motility, we examined whether mutations in Nla28 target genes affected colony spreading on agar surfaces. Specifically, cells carrying mutations in Nla28 target genes were placed on the surface of 1.5% and 0.4% agar plates. The plates were incubated at 32°C for 3 days, and the colony diameters were compared to those produced by wild-type cells, the negative control cells, which were unable to actively spread on agar surfaces due to mutations that inhibit social motility and adventurous motility (i.e., the two motility systems that M. xanthus uses for surface spreading; (34, 35)).

The results indicated that two of the mutations in Nla28 target genes had a strong impact on motility because the strain carrying an insertion in MXAN2510 or MXAN7279 showed a substantial reduction in surface spreading compared to the wild-type strain (Table 3). In the case of the MXAN7279 insertion mutant, a reduction in surface spreading was observed on 0.4% agar plates, which provided a soft and wet surface that favored social motility, but not on 1.5% agar plates, which provided a relatively firm and dry surface that favored adventurous motility (36). For the MXAN2510 insertion mutant, surface spreading on 1.5% agar plates was slightly reduced, and surface spreading on 0.4% agar plates was substantially reduced. This phenotype was reminiscent of the *pilA* (social motility) mutant, which displayed reduced surface spreading on both agar surfaces, but the reduction was most dramatic on 0.4% agar (Table 3).

**TABLE 3:** Swarm diameters of wild-type and mutant cells on 0.4% and 1.5% agar^*a*^

Strain^*b*^	Mean swarm diam (percentage of wild-type)	
	Soft agar (0.4%)	Hard agar (1.5%)
DK1622 (wild-type)	100 ± 10	100 ± 5
DK 2161 (A, S)^*c*^	38 ± 3	37 ± 3
*mrpB* ^*b*^	94 ± 8	100 ± 5
*nla28* ^*b*^	96 ± 5	93 ± 6
*pilA* ^*b*^	27 ± 10	72 ± 6
MXAN881^*b*^	101 ± 10	96 ± 11
MXAN989^*b*^	88 ± 11	81 ± 10
MXAN2510^*b*^	48 ± 3	82 ± 7
MXAN5040^*b*^	89 ± 5	82 ± 4
MXAN6732^*b*^	108 ± 12	108 ± 4
MXAN7147^*b*^	96 ± 7	94 ± 4
MXAN7279^*b*^	41 ± 7	110 ± 3

a The mean diameter (±standard deviation) of five swarms produced by each mutant strain was determined and normalized to the mean diameter of five swarms produced by wild-type strain DK1622.

b The mutant strain of M. xanthus contained an insertion in the indicated gene.

c A, defect in adventurous motility (A-motility); S, defect in social motility (S-motility).

## DISCUSSION

### The likely binding site of Nla28 was an 8-bp direct repeat.

One goal of this study was to better understand how Nla28 identifies its target developmental promoters. Using a collection of potential promoter targets (Table 1), *in vitro* promoter binding assays (Fig. 3), and *in vitro* mutational analyses (Fig. 4), similar 8-bp sequences (consensus CT[C/G]CG[C/G]AG) were implicated in Nla28 binding. Our *in vivo* studies further suggested that the 8-bp sequences (Fig. 5), as well as the Nla28 protein (Table S3 in Supplemental File 1 and Fig. 2), were important for the activity of Nla28 target promoters in developing M. xanthus cells. Assuming Nla28 bound to DNA as a dimer, which would be consistent with other characterized EBPs (23, 24), we proposed that the DNA binding sites of Nla28 were tandem, imperfect 8-bp repeats. Based on scans of the 300 bp upstream of putative −12-bp and −24-bp regions, most of the Nla28 target promoters had clusters of 3 to 5 imperfect repeats (Fig. 1). In fact, seven promoters were predicted to have two such clusters. Thus, most of the promoters had multiple putative sites for the binding of Nla28 dimers. One possible explanation for this arrangement is that the clustering of binding sites helps sequester Nla28 at the promoters, increasing local concentrations. A relatively high local concentration of Nla28 would in turn facilitate transcription, even when the individual binding sites in the promoter are low-affinity sites (37–39).

### Potential Nla28 binding sites were found upstream of many developmental genes.

Because we were interested in getting a more global view of the numbers and types of developmental genes that Nla28 directly regulated, we used the M. xanthus genome sequence, the consensus Nla28 binding site, bioinformatics, and expression data to identify additional promoter/gene targets of Nla28. As shown in Table S5 in Supplemental File 1, an additional 58 putative σ^54^ promoters and 102 genes were tagged as potential targets of Nla28 using the consensus Nla28 binding sequence, bringing the total number of candidates for direct Nla28 regulation to 70 promoters and 140 genes. Interestingly, many of the putative promoters that we identified have multiple tandem 8-bp sites and, hence, multiple potential sites for the binding of Nla28 dimers, which was consistent with the initial 12 promoters that we examined. The types of genes that were discovered in this analysis are discussed below.

### The vast majority of potential Nla28 target promoters were intragenic.

In a recent study of natural product gene regulation in M. xanthus (22), 89% of experimentally confirmed and putative σ^54^-RNA polymerase and Nla28 binding sites were localized to natural product genes and not to intergenic sequences. In our analysis of 12 developmental promoter targets of Nla28, which was described here, we obtained similar results (75% were in protein-coding sequences, as summarized in Fig. 1). This also held for the putative developmental promoter targets of Nla28 listed in Table S5 in Supplemental File 1. With these findings and previous data linking the majority of the σ^54^ promoter targets of Nla6 to intragenic regions (19), a pattern of intragenic σ^54^ promoter usage in M. xanthus started to emerge. However, we are still unable to make sweeping conclusions regarding the genomic locations of M. xanthus σ^54^ promoters due to a lack of experimental data. This aside, it is worth the reminder that *in vivo* mutational analyses have been performed on several intragenic promoter targets of Nla6 and Nla28 (6, 22, 29), and the results indicated that the promoters had the signature properties of σ^54^ promoter elements and were crucial for developmental and/or growth-phase related gene expression. Thus, it seems these intragenic targets of Nla6 and Nla28 are bona fide σ^54^ promoters, as predicted. Given the experimental confirmation of these intragenic promoters, we suggest that having a promoter in the coding sequences of genes might have advantages in some cases. For example, some of these putative σ^54^ promoters were in the coding sequence of a gene that was far upstream of the single gene or operon they were predicted to regulate. This distant location would produce a relatively long 5′ untranslated region (UTR) in the mRNA and, perhaps, the 5′ UTR provided an additional layer of regulation for natural product or developmental gene expression. Other intragenic σ^54^ promoters appeared to be internal, where promoters were located within a gene in an operon instead of upstream of the first gene in the operon. In such cases, the promoter is predicted to yield an mRNA corresponding to a subset of an operon’s genes, providing flexibility if only some of the genes are needed in a particular environment (40).

### Most of the confirmed Nla28 targets had regulatory functions or were predicted to have defense functions.

As noted above, the molecular/cellular functions of some of the confirmed developmental targets of Nla28 were identified before this work. Regulatory or signal transduction functions dominated this group of Nla28 targets because all but one of these loci fell into this category. This included the *actB*, *nla6*, *nla28*, and *mrpB* loci, which contained genes that code for EBPs. These EBPs modulated the transcription of different types of developmental genes (15, 16, 28–30, 41–43) (Table 1). The EBP loci also contained genes for signal transduction proteins such as the histidine protein kinase partners of Nla6 and Nla28, which are called Nla6S and Nla28S, respectively (16, 19–21, 44). The one confirmed and previously characterized Nla28 target that does not have a regulatory function is *pilA*. The *pilA* locus contains genes that are important for type IV pili-based social motility, surface spreading, and normal development (15, 16, 31–33, 45, 46).

**FIG 6:**
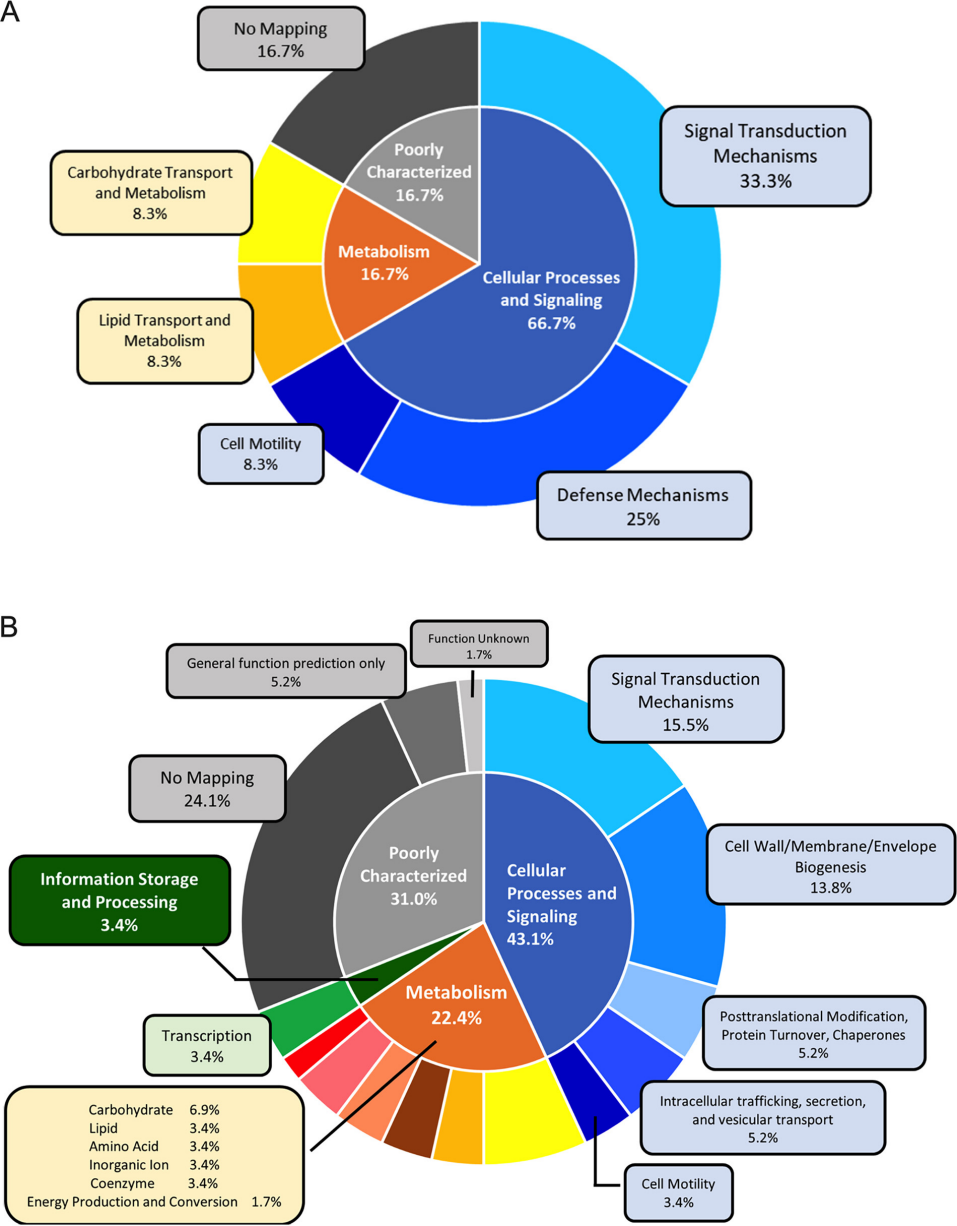
Functional category and subcategory distributions of Nla28 targets based on the clusters of orthologous groups of proteins (COGs) database. (A) The two-layer pie chart shows COG functional categories, subcategories, and their relative abundances of confirmed Nla28 targets in this study. (B) The two-layer pie chart shows COG functional categories, subcategories, and their relative abundances of putative Nla28 targets in this study. The inner layer of pie charts represents categories. The outer layer represents subcategories. The subcategory of no mapping indicates hypothetical proteins without predicted orthologs in the COG database.

Here, we performed a preliminary characterization of the remainder of the confirmed Nla28 targets via mutational analysis. We found that all the loci were important for development, and two were important for surface spreading and, presumably, motility (Table 2 and 3). The most common molecular/cellular functional category assigned to these loci, based on the annotated genome sequence of wild-type strain DK1622 (16), was a defense mechanism (Table S6 in Supplemental File 1 and Fig. 6A). This included genes that were predicted to encode heavy metal efflux transporter components. It also included CRISPR-associated proteins, which have been characterized in a variety of bacterial species and are known to be involved in adaptive immunity against foreign genetic elements (47–51). For the remaining Nla28 target loci characterized here, two were linked to potential metabolic functions. Namely, one locus was predicted to be involved in lipid metabolism and the second in carbohydrate metabolism (Table S6 in Supplemental File 1 and Fig. 6A). The other two loci characterized here had not been assigned a particular function. However, the three Nla28 targets characterized in a recent study (22) are worth mentioning because they are likely involved in the production of secondary metabolites and, hence, could have a defense function, predation function, or be involved in signaling.

It has been suggested that Nla28 is part of a general stress response induced by nutrient depletion because Nla28 modulates the transcription of many early, starvation-responsive developmental genes and genes that are highly expressed during the transition into stationary-phase (6, 21, 22). That some confirmed targets have defense functions makes sense if Nla28 is viewed as a regulator of starvation-induced stress. The metabolic functions of other Nla28 targets also make sense in this context because one would expect nutrient depletion to be accompanied by changes in cellular metabolism. Additional information that supports the view of Nla28 as a regulator of M. xanthus’ stress response comes from physiological studies in other Gram-negative bacteria. Orthologues of MXAN881, MXAN989, MXAN5040, MXAN7147, and MXAN7280 have all been linked to stress responses in other Gram-negative bacteria (52–56) (Table S6 in Supplemental File 1 and Fig. 6A).

### Regulatory, metabolic, and cell envelope biogenesis were common functions among putative Nla28 targets.

As noted above, 58 putative σ^54^ promoters and 102 genes were tagged as potential targets of Nla28 using the consensus Nla28 half-binding site, bringing the total number of candidates for direct Nla28 regulation to 70 promoters and 140 genes. Based on the annotated genome sequence of wild-type strain DK1622 (16), we were able to place the putative Nla28 targets into molecular/cellular functional categories (Table S7 in Supplemental File 1 and Fig. 6B). The three most highly represented categories were metabolic functions, regulatory/signal transduction, and cell envelope/cell wall biogenesis. Given that developing cells were experiencing starvation, changes in cellular metabolism might be expected. Similarly, developing cells might be expected to express genes involved in the cell wall or cell membrane structural changes because developing cells were experiencing starvation-induced stress. Other notable categories of putative Nla28 targets included motility genes, posttranslational modifications/protein turnover, and protein secretion. Finally, many of the putative Nla28 targets had predicted orthologues linked to stress responses in other Gram-negative bacteria. For example, potential orthologues of MXAN162, MXAN934, and MXAN2907 have been linked to envelop stress in Gram-negative species (57–60) (Table S7 in Supplemental File 1 and Fig. 6B).

In conclusion, Nla28 is an EBP that began modulating gene expression soon after M. xanthus cells encounter nutrient-poor conditions. In this study, we identified the direct targets of Nla28 to better understand Nla28’s function and to help define the early gene regulatory pathways involved in M. xanthus’ starvation response. Our data suggest that Nla28 might recognize its target promoters using tandem, imperfect repeats of an 8-bp sequence and that most of the Nla28 target promoters are intragenic. Seventy promoters and 140 genes have been classified as potential targets of Nla28, suggesting that the Nla28 regulon might be relatively large. Some of the functions assigned to Nla28 target genes are regulatory, metabolism, defense-related functions, and cell envelope biogenesis. Many of these functions make sense in the context of Nla28’s role as a general regulator of stress-associated genes and, based on the work presented here, we now know that several of these genes are important for the production of stress-resistant spores following starvation.

## MATERIALS AND METHODS

### Bacterial strains and plasmids.

The strains, plasmids, and primers used in this study are shown in Table S1 and S2 in Supplemental File 1. Plasmids generated for this study were analyzed via DNA sequence analysis. M. xanthus strains were confirmed as described below. Plasmid insertions in Nla28 target genes in wild-type strain DK1622 were generated as previously described (15, 61) and confirmed via PCR and DNA sequencing.

### Growth and development.

Escherichia coli strains were grown at 37°C in LB broth containing 1.0% tryptone, 0.5% yeast extract, and 0.5% NaCl or on plates containing LB broth and 1.5% agar. LB broth and LB plates were supplemented with 40 μg of kanamycin sulfate/mL, 100 μg of ampicillin/mL, or 10 μg of an oxytetracycline/mL as needed.

M. xanthus strains were grown at 32°C in CTTYE broth (1.0% Casitone, 0.5% yeast extract, 10 mM Tris-HCl [pH 8.0], 1 mM KH_2_PO_4_, and 8 mM MgSO_4_) or on plates containing CTTYE broth and 1.5% agar. CTTYE broth and plates were supplemented with 40 μg of kanamycin sulfate/mL or 10 μg of an oxytetracycline/mL as needed.

Fruiting body development was induced by placing M. xanthus cells on plates containing TPM buffer (10 mM Tris-HCl [pH 8.0], 1 KH_2_PO_4_, and 8 mM MgSO_4_) and 1.5% agar or in 6-well microtiter plates containing MC7 buffer (10 mM MOPS, 1 mM CaCl_2_, final pH 7.0), and incubating the plates at 32°C. Briefly, M. xanthus strains were grown in flasks containing CTTYE broth, and the cultures were incubated at 32°C with vigorous swirling. The cells were pelleted when the cultures reached a density of about 5 × 10^8^ cells/mL, the supernatants were removed, and the cells were resuspended in TPM buffer or MC7 buffer to a final density of 5 × 10^9^ cells/mL. Aliquots of the cells in the TPM buffer were spotted onto TPM agar plates and aliquots of the cells in the MC7 buffer were placed in 6-well microtiter plates containing MC7 buffer. The cells were incubated at 32°C and development was monitored as previously described (15, 19). Cells were harvested at various times during development and prepared for quantitative PCR (qPCR), β-galactosidase assays, or sporulation assays.

To examine the sporulation efficiency of each M. xanthus strain, developing cells were harvested from TPM agar plates after 5 days and the cells were placed in 400 μL of TPM buffer. The resuspended cells were first dispersed by a 10 s burst of sonication using a model 100 Sonic Dismembrator (Fisher Scientific) that was set at an intensity of 1.5. Aliquots of the dispersed cells were placed in a Petroff-Hausser counting chamber, and phase-contrast microscopy was used to determine the number of spherical-shaped cells that were present. Other aliquots of the cell suspension were subjected to three 10 s bursts of sonication using an intensity setting of 4, and the sonication-treated cells were incubated at 50°C for 2 h. The number of heat- and sonication-resistant spores that germinated into colonies was determined by placing heat- and sonication-treated cells in liquified CTT soft agar (1.0% Casitone, 10 mM Tris-HCl [pH 8.0], 1 mM KH_2_PO_4_, 8 mM MgSO_4_, and 0.7% agar), pouring the soft agar onto CTTYE agar plates and incubating the plates at 32°C for 5 days.

### Motility assays.

Motility assays were performed as previously described (15, 61). Briefly, M. xanthus cells were grown to a density of about 5 × 10^8^ cells/mL in CTTYE broth. The cells were pelleted by centrifugation, the supernatant was removed, and the cells were resuspended in CTTYE broth to a density of 5 × 10^9^ cells/mL. Aliquots (3 μL) of the cell suspensions were spotted onto CTTYE plates containing 1.5% or 0.4% agar, the spots were allowed to dry, and the plates were placed at 32°C. After 3 days of incubation, five swarms of each strain were measured, and their mean diameter was normalized to the mean diameter of five swarms formed by wild-type strain DK1622.

### Plasmid transfer to M. xanthus.

Plasmids containing internal fragments of Nla28 target genes or wild-type or mutant copies of the *nla28* promoter were electroporated into wild-type strain DK1622 as described previously (62). Kan^r^ electroporated that contained a plasmid integrated into the *nla28* locus or in a *nla28* target gene by homologous recombination or that contained a plasmid integrated into the chromosomal Mx8 phage attachment site (*attB*) by site-specific recombination were identified via PCR and DNA sequencing. Kan^r^ electroporated carrying a single plasmid insertion were assayed for development, motility, or promoter activity as needed.

### Standard DNA procedures.

Chromosomal DNA from M. xanthus strains was extracted using a ZYMO Research gDNA Extraction kit. Oligonucleotides used in PCRs were synthesized by Integrated DNA Technologies (IDT) and are listed in Table S2 in Supplemental File 1. Plasmid DNA was extracted using the Promega Nucleic Acid Purification kit. Amplified and digested DNA fragments were purified using the Gel Extraction Minipreps kit from Bio Basic. For all kits, the manufacturer’s protocols were used. The compositions of all plasmids and promoter fragments were confirmed by DNA sequencing (Genewiz).

### *In vivo* mutational analysis of the putative Nla28 binding site in the *nla28* promoter.

Wild-type and mutant *actB*, *nla28*, *pilA*, and MXAN5040 promoter fragments were cloned into the promoterless *lacZ* expression vector pREG1727 to create *lacZ* transcriptional fusions (27). The plasmids were introduced into strain DK1622 or a derivative of strain DK1622 carrying an insertion in the *nla28* gene, and cells carrying a plasmid integrated at the Mx8 phage attachment site in the chromosome were identified via PCR and DNA sequence analysis. The *in vivo* activities of wild-type and mutant promoters were determined by measuring the specific activities of β-galactosidase in cells developing in submerged cultures for 1, 2, 6, 8, 12, 24, or 48 h (22, 26). The β-galactosidase-specific activity was defined as nanomoles of o-nitrophenol (ONP) produced per minute per milligram of protein. Wild-type and mutant promoters were analyzed in triplicate at each time point using independent biological samples.

### Expression and purification of Nla28-DBD.

A fragment of the *nla28* gene corresponding to the Nla28 DNA binding domain (Nla28-DBD) (6, 22) was PCR amplified using gene-specific primers (Table S2 in Supplemental File 1) and then cloned into plasmid pMAL-c5x to create an N-terminal maltose binding protein (MBP) fusion to Nla28-DBD. The Nla28-DBD expression plasmid was introduced into E. coli strain BL21(DE3) using electroporation. Cells containing Nla28-DBD expression plasmids were grown in rich LB broth to a density of approximately 2 × 10^8^ cells/mL. Protein expression was induced by the addition of 0.3 mM Isopropyl β-D-1 thiogalactopyranoside (IPTG) to the culture (500 mL to 1 L) and the subsequent incubation of the culture for 12 h at 15°C. Cells were pelleted via centrifugation and resuspended in 25 mL column buffer (20 mM Tris-HCl, 200 mM NaCl, 1 mM EDTA, 5 U/mL DNase I, 1 mM DTT, pH 7.4) per L of culture. The resuspended cells were lysed by a combination of freeze-thawing and sonication and pelleted by centrifugation. The crude extract (supernatant) containing Nla28-DBD was diluted by adding 125 mL of cold column buffer to every 25 mL aliquot of crude extract. For each purification, 100 mL of diluted crude extract containing Nla28-DBD was loaded onto 5 mL MBPTrap HP columns (GE Healthcare) at a flow rate of 5 mL/min and washed with 600 mL cold column buffer at a flow rate of 10 mL/min on an ÄKTA Fast Protein Liquid Chromatography (FPLC) system (GE Healthcare). MBP-tagged Nla28-DBD was eluted using a 100 mL cold column buffer containing 10 mM maltose. The flow rate was 5 mL/min, and 20 fractions containing 5 mL were collected. The presence and concentration of eluted fusion protein were detected by UV absorbance at 280 nm. Nla28-DBD-containing fractions were pooled and incubated with 1 mg of Factor Xa per 100 mg of fusion protein at 4°C overnight to cleave the MBP tag. Nla28-DBD was separated from MBP and concentrated to about 1 mg/mL using 30 kDa and 10 kDa Amicon Ultra centrifugal filter units (EMD Millipore). SDS-PAGE (Fig. S1 in Supplemental File 1) and Bradford assays were used to determine the purity and concentration of Nla28-DBD.

### Electrophoretic mobility shift assays.

The PCR-generated fragments of the putative Nla28 target promoters contained approximately 180 to 220 bp of DNA upstream of the σ^54^-RNA polymerase binding sites, which were identified experimentally (6, 19, 26, 63) or using a bioinformatics tool (PromScan) that was specifically developed to find such sites in the sequences of bacterial DNA (25). For use in electrophoretic mobility shift assays, the promoter fragments were PCR amplified using 5′Cy5-labeled primers synthesized by IDT (Table S2 in Supplemental File 1). Binding reactions contained EMSA buffer (25 mM Tris/acetate [pH 8.0], 8.0 mM magnesium acetate, 10 mM KCl, 1.0 mM DTT), 2.0 μM Nla28-DBD and 5 ng of a 5′ Cy5-labeled promoter fragment in a total volume of 10 μL. The binding reactions were allowed to proceed for 30 min at 30°C, and EMSAs were performed under 100 V for 60 min using native (nondenaturing) PAGE (3% to 12%, Bis-Tris, Invitrogen). The binding reactions were imaged and analyzed by a Bio-Rad imager.

### Quantitative PCR.

To examine the expression of Nla28 target genes, wild-type cells, and cells with an inactivated *nla28* gene were harvested during vegetative growth (0 h) and development in submerged cultures. Total cellular RNA was isolated from developmental cells using the RNAprotect Bacteria Reagent (Qiagen) and the RNeasy Minikit (Qiagen) as described in the manufacturer’s protocols. To help lyse developmental cells, 0.1 mm diameter glass beads were added after the lysis buffer and the cell suspensions were subjected to vigorous shaking using a VWR DVX-2500 multitube vortex. For each time point (0, 1, 2, 8, 12, or 24 h), total RNA was isolated from seven independent biological replicates of the wild-type strain and seven independent biological replicates of the *nla28* mutant. The wild-type RNA samples from each time point were pooled and the *nla28* mutant RNA samples from each time point were pooled, and the pooled samples were subsequently used to generate cDNA as previously described (19, 44, 64). The CFX Connect real-time PCR detection system (Bio-Rad) was used to perform the qPCR analysis (19). Each time point for the wild-type and *nla28* mutant was analyzed in triplicate (i.e., we analyzed three technical replicates of each pooled sample). Relative fold changes in mRNA levels were calculated using the reference gene *rpoD* or 16S rRNA and the ΔΔCT method as previously described (19, 44).

### Statistical analysis.

Individual sample sizes are specified in each figure legend. Comparisons between groups were assessed by two-way analysis of variance (ANOVA) with Tukey’s multiple comparisons *post hoc* tests, as appropriate. The significance level was set at *P* < 0.05 or lower. Prism (GraphPad) v9.2 was used for all analyses.
